# Knowledge of Dietary Supplements and Attitudes Towards Complementary Medicine Among University Students: A Cross-Sectional Study

**DOI:** 10.3390/foods15010061

**Published:** 2025-12-24

**Authors:** Sara Bezak, Ksenija Baždarić, Lea Huzjak Horvat, Darko Lončarić, Vanja Brandić Mičetić, Sabina Fijan, Maja Šikić Pogačar, Sandra Kraljević Pavelić

**Affiliations:** 1 Clinical Hospital Center Rijeka, 51000 Rijeka, Croatia; bezak.sara@gmail.com; 2 Faculty of Health Studies, University of Rijeka, 51000 Rijeka, Croatialea@svoya.hr (L.H.H.); 3 The Faculty of Teacher Education, University of Rijeka, 51000 Rijeka, Croatia; dloncaric@ufri.uniri.hr (D.L.); vanja.brandic1@uniri.hr (V.B.M.); 4 Faculty of Health Sciences, University of Maribor, 2000 Maribor, Slovenia; sabina.fijan@um.si; 5 Faculty of Medicine, University of Maribor, 2000 Maribor, Slovenia; maja_sikic@yahoo.com.au

**Keywords:** dietary supplements, complementary and alternative medicine, university students, knowledge, attitudes

## Abstract

Despite the increasing global consumption of dietary supplements (DS) and the growing interest in complementary and alternative medicine (CAM), there is a significant gap in evidence-based knowledge and understanding of these practices among university students, particularly those in non-health-focused programs. This gap may lead to misconceptions, misuse, and unsafe practices, which necessitate targeted educational interventions. The presented cross-national comparative study assessed knowledge of DS and attitudes towards CAM among 809 university students from Croatia and Slovenia, including health-focused and non-health-focused study programs by use of validated questionnaires. The study integrated DS and CAM within the same analytical framework. Assessed knowledge on DS was moderate, with an average of 71.2% correct answers. Slovenian students from health-focused studies achieved the highest scores, while Croatian students from non-health focused studies scored the lowest values. Misconceptions persisted across all groups, while usage of supplements was widespread. Attitudes toward CAM were overall mildly positive, where Slovenian students from health-focused studies reported the most favorable views. Attitudes were more strongly associated with supplement use than with knowledge, indicating that personal experience and cultural context shape perceptions more than formal education. Our findings challenge the usual assumption that higher knowledge automatically leads to rational health decisions.

## 1. Introduction

In recent decades, global consumption of dietary supplements (DS) has increased, driven by advancements in manufacturing, expanded regulatory frameworks, growing public demand and market dynamics that reflect the advances in scientific knowledge on this group of products [1,2]. Dietary (nutritional) supplements encompass a wide range of products such as nutraceuticals and, due to their clinical benefits, may play an important role in modern medicine as well; DS are intended to complement the usual diet and maintain health by providing concentrated sources of vitamins, minerals, or other bioactive compounds [3,4,5]. DS are available in various forms, such as capsules, tablets, pastilles, sachets with powder, and concentrates in vials, and they can be individual or multi-compound preparations, which facilitate and simplify their consumption [6,7]. Commonly used supplements in the general population include B-complex vitamins, fat-soluble vitamins, minerals such as potassium, zinc, magnesium, selenium, and antioxidants such as, for example, vitamin E, vitamin C, quercetin, curcumin, resveratrol, and creatine [3,8,9,10,11,12,13,14,15,16,17,18]. Studies have shown that while athletes are among the most frequent users, with the aim of increasing the performance level, approximately 50% of U.S. adults consume DS for health maintenance or to prevent nutritional deficiencies [19,20,21,22,23,24].

Although they are classified as food products and are now used for therapeutic purposes or a balanced diet replacement, the role of DS is increasingly acknowledged in preventing and managing micronutrient deficiencies, supporting nutritional adequacy in at-risk groups, and addressing conditions such as osteoporosis, anemia, and cardiovascular disease through targeted supplementation, where supplements such as vitamin D, iron, folic acid, calcium, and omega-3 fatty acids have demonstrated their therapeutic value [25,26,27,28]. The National Institutes of Health (NIH) published consumer guidelines promoting a rational evaluation of the true needs for and knowledge about a specific food supplement’s clinical effect [29]. DS consumption is also linked to complementary and alternative medicine (CAM), which combines non-conventional diagnostic, preventive, and treatment approaches, with or instead of standard care. Furthermore, along with the increased usage of DS in the general population, there is a growing interest in the use of complementary and alternative medicine. According to some studies, CAM practices are highly prevalent worldwide, reported by 70–90% of adults in Europe, 36–38% in the U.S. (CAM), and 55% in Croatia [30,31,32,33,34,35,36,37]. This trend highlights the increasing importance of understanding CAM practices and their integration into modern healthcare, especially in the post-COVID period when their application came intensively into the healthcare spotlight, underlying the need for robust regulatory frameworks to ensure safety, accurate labeling, and timely reporting of clinical trial data [2,38,39]. European surveys, for example, demonstrate the variable inclusion of DS in national nutrition monitoring, reflecting ongoing gaps in public health oversight [38]. Together, these findings support the need to update curricula and regulations in line with evolving scientific evidence and societal trends.

Accordingly, the presented study aimed to assess knowledge of DS and attitudes towards CAM among 809 university students from Croatia and Slovenia, including health-focused and non-health-focused study programs by use of validated questionnaires. By identifying knowledge gaps and attitude patterns, the study aims to provide evidence for curriculum development and educational interventions to improve health literacy and promote safe, evidence-based practices.

## 2. Materials and Methods

### 2.1. Participants

Students of health-focused sciences (Faculty of Health Studies, University of Rijeka, Croatia and Faculty of Health Sciences in Maribor, University of Maribor, Slovenia) and students from the field of education (undergraduate studies in Early and Preschool Education from the Faculty of Teacher Education in Rijeka, University of Rijeka, Croatia) participated in the research. Students who received any kind of formal education in nutritional sciences (e.g., students majoring in clinical nutrition) were excluded from the study.

### 2.2. Procedure

For the purposes of this research, two questionnaires for the assessment of DS knowledge and CAM attitude evaluation were used within the same analytical framework. Filling out the questionnaire was anonymous, voluntary, and required 5–10 min, and the research itself was conducted through the period from May 2025 to June 2025. The research included all adult male and female students who gave their consent to participate.

### 2.3. Questionnaires

#### 2.3.1. Assessment of Knowledge About Dietary Supplements

The first questionnaire, “Knowledge about dietary supplements” [40], was translated from English (Supplementary Material S3) to Croatian and Slovenian (Supplementary Material S1 and S2), using a standard double-blind procedure. The questionnaire contains 17 questions based on the true-false principle and was used to measure students’ knowledge about the impact of dietary supplements on health. One point was awarded for each correct answer. Based on the number of correctly answered questions, students were ranked according to their level of knowledge, from “unsatisfactory” (“failed test”—the number of correctly answered questions was less than 9), “passed” test (the number of correctly answered questions was >=9), and “excellent level of knowledge” (the number of correctly answered questions was 15–17).

The variable percentage of correct answers is a linear composition of the number of correct answers divided by 17 and multiplied with 100, with a range from 0–100.

#### 2.3.2. Assessment of Attitude Towards CAM

The second questionnaire, “Attitude towards CAM” [41] (Supplementary Material S1–S3), was shortened with the permission of the author for the purposes of this research. The questionnaire consists of three parts.

The first part included the socio-demographic characteristics of the respondents (age, gender, and field of study).

The second part is related to the frequency of using CAM methods according to the assessment scale, from 1 (“never”) to 5 (“very often”)—it included 31 CAM methods. Variable usage of supplements includes 4 items (herbs, vitamins and minerals, probiotics, and other supplements), and the total results are a linear composition divided by the number of items with a total range of 0–4.

The third part of the questionnaire was used to measure trust and willingness to use CAM methods, using a Likert scale for evaluation from 1 (“completely disagree”) to 5 (“completely agree”), originally with 10 items. The final variable, “Attitude towards CAM”, has 7 items and the total result is a linear composition divided by the number of items, with a total range of 1–5. Items 2, 5, and 7 were recorded and questions 4, 8, and 9 were excluded from the total score (items 4 and 8 were not adapted for teachers and item 9 had a low correlation with the total score). Lower results (<2.6) were considered to be a negative attitude, average (2.6–3.39) to be a neutral attitude, and higher results (>3.39) to be a positive.

### 2.4. Hypotheses of the Study

**H1:**  *Health-focused students will demonstrate significantly higher knowledge about DS compared to non-health-focused students, due to curriculum exposure to nutrition and pharmacology.*

**H2:**  *Health-focused students will exhibit more positive attitudes toward CAM than non-health-focused students, reflecting greater familiarity with integrative health approaches.*

**H3:**  *Knowledge about DS will positively correlate with attitudes toward CAM, assuming that education fosters evidence-based acceptance.*

**H4:**  *Supplement usage will positively correlate with attitudes toward CAM, as personal experience shapes health-related beliefs more than formal knowledge.*

### 2.5. Statistical Analysis

Categorical data are shown as frequency and relative frequency and tested with χ2 test. Quantitative data are presented as mean and standard deviation and differences between faculties were tested with one-way ANOVA and a post hoc Newman–Keuls test. The reliability of attitude towards CAM was calculated as internal consistency coefficient Cronbach alpha. Correlation was assessed with the Spearman correlation coefficient. *p* < 0.05 value was considered to be statistically significant. Jamovi version 2.3.28.0. (https://www.jamovi.org; Sydney, Australia) was used for statistical analysis.

## 3. Results

### 3.1. Characteristics of the Participants

Altogether, there were 809 participants from two countries and three faculties and the data are presented in Table 1. The participants were on average 23.9 (SD = 7.4) years old, mostly bachelors (n = 690 (79.7%)).

### 3.2. Knowledge About Dietary Supplements

Knowledge about dietary supplements is presented in Supplementary Material S4. In total, the average score of the test for all participants was 71.2 (14.0)%. There was a significant difference in the percentage of correct answers between the faculties involved in the study (*p* = 0.005): the highest percentage was observed for the health-focused study in Croatia (M = 72.4 (SD = 13.2)) and the lowest for the non-health focused study in Croatia (M = 68.8 (SD = 14.1), and those two groups differed significantly in the post hoc Student–Newman–Keuls test (S4). The result for the related health-focused faculty in Slovenia (M = 71.8 (SD = 17.1)) was comparable with the result obtained for the health-focused study in Croatia (Supplementary Material S4).

Only 7% of participants did not pass the test and there was no significant difference between the involved faculties (*p* = 0.167). We have additionally tested the gender differences in the variable test pass and no differences were established (*p* = 0.335). However, there was a significant difference in grade distribution (*p* = 0.010) and the health-focused study in Slovenia had significantly more high grades (13.8%) than the other two faculties in Croatia (6.3% in health studies and 5% in education studies (Supplementary Material S4).

The most difficult question for the students were as follows: Q5 (dietary supplements are food) with n = 96 (11.9%) correct answers, followed by Q12 (the use of multivitamin preparations protects against heart diseases)—n = 219 (27.2%) and Q13 (the use of antioxidants prevents the development of cancer)—n = 280 (34.7%). The easiest question was Q1 (before being marketed, dietary supplements must be tested for efficacy and safety), with n = 794 (98.1%) correct answers. There were significant difficulties between faculties in Q3 (the quality of dietary supplements is routinely tested before being marketed), Q4 (the packaging of dietary supplements must contain information on possible adverse effects resulting from their use), Q5 (dietary supplements are food), Q9 (in the elderly, taking vitamin D reduces the risk of bone fractures), Q11 (taking dietary supplements containing calcium reduces the risk of bone fractures in the elderly), Q12 (the use of multivitamin preparations protects against heart diseases), Q13 (the use of antioxidants prevents the development of cancer), and Q14 (regular use of vitamin C reduces the risk of catching a cold).

### 3.3. Usage of Herbs, Vitamins and Minerals, Probiotics, and Other Supplements

**Figure 1:**
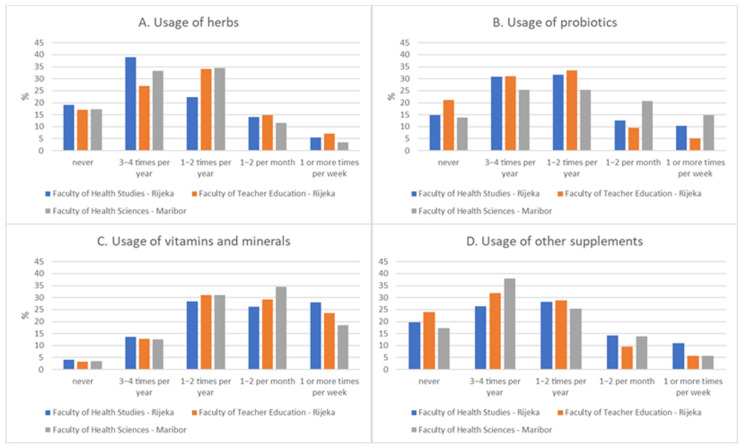
Relative frequency (%) of: usage of herbs, (**A**), probiotics, (**B**), vitamins and minerals, (**C**), and other supplements, (**D**).

The relative frequency of usage of herbs, vitamins and minerals, probiotics, and other supplements is presented in Figure 1. We have found differences in usage of herbs (*p* = 0.014), probiotics (*p* < 0.001), and other supplements (*p* = 0.043). Herbs are most often used by those studying education in Croatia (7.1%), then health studies in Croatia (5.5%) and Slovenia (3.4%). There was no difference (*p* = 0.641) in usage of vitamins and minerals and those are consumed most often by the students of health studies in Croatia (28%), education study in Croatia (23.6%), and health studies in Slovenia (18.4%).

Probiotics are most often used by those studying health studies in Slovenia (14.9%), Croatia (10.3%) and least by those studying education in Croatia (5.8%). Finally, other supplements are most often used by those studying health studies in Croatia (10.9%), those studying education in Croatia (5.8%) and those studying health studies in Slovenia (5.7%).

### 3.4. Attitude Towards CAM

**Figure 2:**
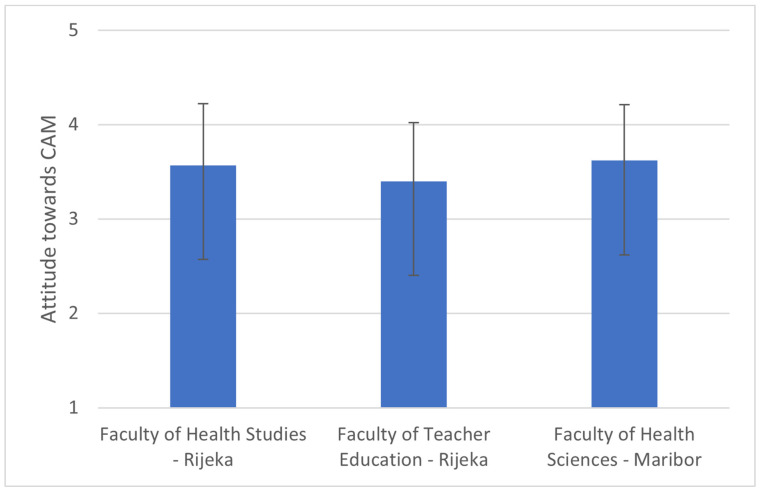
Differences in attitude towards CAM, according to the faculty. Lower results (<2.6) were considered to be a negative attitude, average (2.6–3.39) to be a neutral attitude, and higher results (>3.39) to be positive.

The attitude towards CAM scale reliability was high: Cronbach α = 0.84. The attitude towards CAM was mildly positive and it differed across the faculties (*p* = 0.002); it was highest in health-focused studies Slovenia [M = 3.62 (SD = 0.59)], followed by Croatia [M = 3.57 (SD = 0.65)], and the lowest score was in the non-health education studies in Croatia [M = 3.40 (SD = 0.62)] (Figure 2). Post hoc Student–Newman–Keuls tests for all pairwise comparisons have shown that all groups were different at a level of *p* < 0.05.

### 3.5. Correlations of Test Results, Usage of Supplements, and Attitude Towards CAM

The correlations between test results and usage supplements were not found, except in the Faculty of Health Studies—Rijeka, where the correlation was positive but very weak (r_S_ = 0.10) (Table 2).

The correlation between test results and attitude towards CAM was significant and positive for the whole sample and the Faculty of Teacher Education was also weak (r_s_ = 0.12 and r_s_ = 0.13). The correlation between the usage of supplements and attitude towards CAM was higher, positive, and significant in all groups, with the highest being in health studies in Slovenia (r_s_ = 0.38) (Table 2).

## 4. Discussion

### 4.1. Knowledge of Dietary Supplements

To the best of our knowledge, this is the first cross-national comparative study between health-focused and non-health-focused studies that simultaneously examined knowledge of DS and attitudes towards CAM among university students from Croatia and Slovenia. The first hypothesis of the study has been supported by the obtained results, as health-focused students demonstrated significantly higher knowledge than non-health students (72.4% vs. 68.8%, *p* = 0.005), confirming the impact of health-related curricula. The difference between Slovenian and Croatian health studies is probably due to a more systematic integration of public health promotion and evidence-based health education into the university curricula of the health-focused programs in Slovenia in comparison to Croatia (see study limitations for further details on socio-cultural differences between these two countries).

The overall knowledge of DS in the presented study was moderate, with a mean score of 71.2% correct answers. Only 7% of participants failed the test. Knowledge gaps were most pronounced for Q5 (dietary supplements are food) (11.9%), Q12 (the use of multivitamin preparations protects against heart diseases) (27.2%), and Q13 (the use of antioxidants prevents the development of cancer) (34.7%), whereas Q1 (before being marketed, dietary supplements must be tested for efficacy and safety) had the highest correct response rate (98.1%). These results align with international evidence showing that knowledge deficiencies persist even among health-related students [42,43,44,45,46]. The obtained results are aligned with rather low factual knowledge about DS previously assessed in studies from Saudi Arabia, the UAE, Jordan, and the United States [34,40,42,43,44,45,47,48,49,50,51,52,53]. However, we want to state that reported ‘knowledge gaps’ may reflect lower-order recall, rather than deficiencies in critical thinking or clinical reasoning, due to the primarily used basic true/false type of questions. In future studies, open-ended questions may, for example, be used to better evaluate applied knowledge and decision-making skills.

Previous research conducted in Croatia and other countries suggests that students frequently use DS and CAM but often lack evidence-based knowledge, potentially leading to misuse, overdosing, or reliance on unproven therapies [34,41,46,50,54,55,56]. Furthermore, most existing studies focus on medical or pharmacy students, while data on students of other health-focused and non-health-focused studies are missing, despite their influence on public health literacy and attitudes toward evidence-based practices [35,36,42,48,57]. This is especially relevant for students of the health studies that are trained to interact often and directly with patients in their future careers. A similar observation may be made from a similar national-based study that was conducted among Iranian students on CAM attitudes between medical and non-medical students, which also showed higher positive attitudes in medical students that the authors correlated with their cultural context and a higher rate of consultations with healthcare professionals [58]. The findings of the presented study may thus contribute directly to informing curriculum development and targeted educational interventions to promote safe and evidence-based use of DS and CAM [45,53,59]. For example, research has demonstrated that implementing structured educational interventions can significantly enhance students’ knowledge and attitudes about dietary supplements and CAM. A study by Sadeghi et al. [60] found that Iranian medical students demonstrated highly variable CAM knowledge (ranging from 12% for homeopathy to 90% for acupuncture), yet 49% maintained positive attitudes and expressed willingness to train. These parallel patterns led Sadeghi et al. to conclude there is ‘a necessity to integrate complementary and alternative medicine into the medical curriculum. Jahan et al. [61] reported that three week elective CAM rotations integrating both lectures and direct experiential learning (shadowing) significantly improved both student attitudes and knowledge, demonstrating that combined didactic–experiential approaches successfully bridge the gap between positive perceptions and evidence-based understanding. The most important finding in this field is provided by Geldenhuys et al. [62], who documented a seven year evolution of a natural product course, showing improvements in student knowledge, counseling skills, and the ability to evaluate the scientific literature. Their intervention combined didactic teaching with clinical case studies, site visits, and interactive sessions—approaches directly addressing the knowledge–attitude gap we identified. Our proposal similarly reflects documented educational needs: the weak knowledge–attitude correlation and stronger usage–attitude correlation empirically demonstrate that current educational approaches are insufficient, necessitating evidence-based pedagogical reform.

### 4.2. Usage Patterns of Dietary Supplements

Usage differed by supplement type and faculty. Herbs were most commonly used by non-health-focused studies students in Croatia (7.1%), probiotics by health-focused studies students in Slovenia (14.9%), and vitamins/minerals primarily by health-focused studies students in Croatia (28%). Other supplements were most frequently used by health-focused studies students in Croatia (10.9%). Similar international patterns are observed: 71% of female health sciences students in Pakistan reported DS use [50], 71% of Saudi students used DS [51], and 74% of adults in Iraq used herbal medicines [63]. Across countries, women typically report higher DS usage and awareness [37,45,50,63]. The usage frequency of DS is broadly similar across countries, but the types of supplements vary, which may be attributed to cultural and educational factors.

### 4.3. Attitudes Toward Complementary and Alternative Medicine

Our second hypothesis was also supported by the obtained results, as health-focused studies students showed more positive CAM attitudes than non-health studies students (3.57–3.62 vs. 3.40, *p* = 0.002), validating the role of educational exposure. Correlations between DS knowledge and CAM attitudes were weak but significant (rs = 0.12–0.13), while correlations between supplement usage and CAM attitudes were stronger (rs = 0.38 in health-focused studies). This indicates that personal experience with DS may influence attitudes more than formal knowledge, echoing findings from U.S. pharmacy students and international surveys [44,52]. This trend is also aligned with recent discussions on the need for personalized medicine advancements, where the patient and the patient’s own experience strongly inform medical decisions [64]. Our study demonstrated that students generally expressed mildly positive attitudes toward CAM, which is consistent with findings from Saudi Arabia [48], Jordan [46], and the United States [52], where positive perceptions persisted despite limited factual knowledge. Comparing the obtained results with the results of the research in the countries of the Middle East, there is a difference in the methods of mind–body practice, where more advantages are given to natural methods and faith and methods that are deeply rooted in ancient beliefs, which are probably related to the cultural environment [64,65,66].

The differences observed across the tested faculties in our sample—highest in health-focused studies students from Slovenia and lowest in the non-health-focused studies students in Croatia—suggest that exposure to health-related education may foster more favorable attitudes: a trend also reported in other medical student cohorts. Importantly, we found that students’ attitudes were more strongly correlated with personal supplement use than with knowledge scores, supporting previous research [42,43] that highlights experience and peer influence as key drivers of attitudes toward CAM. The high internal consistency of the CAM attitude scale in our study (Cronbach α = 0.84) further strengthens the validity of these findings. Collectively, these results indicate that while formal education may enhance awareness, attitudes appear to be shaped primarily by behavioral and cultural factors, rather than by factual understanding. Accordingly, our third and fourth hypotheses were only partially supported, suggesting that education alone is insufficient to shape attitudes and that experiential learning dominates attitude formation. Such findings underscore the need for structured curricula that not only provide knowledge but also critically address perceptions and beliefs about CAM to ensure that future professionals adopt evidence-based approaches.

Although many studies show a positive attitude of the medical staff towards CAM methods and the use and wide acceptance of the CAM method is increasing among medical professionals [67,68], this research shows that the highest attitude is shown by students of Midwifery and Physiotherapy, while the lowest attitude is shown by Nursing students. This result may be due to the fact that certain types of CAM methods, such as aromatherapy, massage, and breathing techniques are often used in the field of midwifery to facilitate the birth process, which is why a more positive attitude towards these methods was recorded. Again, the positive trend correlates with personal experience. On the other hand, due to stricter protocols and rules imposed by the western healthcare system, the more negative attitude of Nursing students towards CAM methods compared to students of other study majors is also visible. This may be related to many factors, such as, for example, a lack of education on the topic, a lack of opportunities for usage in everyday practice, or other personal attitudes. Indeed, within the curriculum for students majoring in Midwifery and Physiotherapy, there is a significantly larger teaching content that includes specific CAM methods, and the practical experience that the students gain during their education and professional practice may also contribute to the more positive attitude observed.

In summary, health students may have more positive attitudes toward CAM because their education and clinical exposure increase awareness of holistic approaches and patient-centered care. In addition, their knowledge of disease and therapy provides them with a good basis for an evidence-based evaluation of CAM’s benefits or limitations. It has to be stated that the integrative medicine topic is growing in the media, incorporating cultural norms and traditional practices. This additionally reinforces familiarity and trust in CAM. We may state accordingly that professional education and socio-cultural influences nurture positive attitudes toward CAM among health students.

### 4.4. Cross-Study Integration and Implications

International studies demonstrate convergent findings: DS/CAM are widely used despite insufficient knowledge [44,45,50,63], attitudes are predominantly positive and influenced by personal experience [44,45,46,55,65], women report higher usage [45,50,63], informal sources dominate information acquisition [45,46,57], and educational interventions improve outcomes [45,48,53,56,59]. In particular, our overall score of 71.2% correct answers (knowledge level) is notably higher than that of US pharmacy students (~50%) [43,52], which is comparable to moderate knowledge in UAE students [57], and superior to the poor knowledge reported in Jordan [46]. However, knowledge gaps persist across all cohorts, particularly regarding safety and interactions. Usage patterns show considerable variation: our vitamins/minerals consumption (18.4–28%) is lower than in Pakistan (71% overall DS use) [50] and Saudi Arabia (71%) [51], yet our probiotic usage (5.8–14.9%) appears to be unique, possibly reflecting European dietary trends. Attitudes toward CAM in our sample (3.40–3.62, mildly positive) mirror the generally positive attitudes reported across most previously published studies [42,44,45,50,51,57], with the exception of Jordan [46] showing negative/neutral attitudes. Critically, our study did not assess information sources which represents a limitation, as information sources strongly influence both knowledge accuracy and attitudes. The cross-national consistency in positive attitudes, despite variable knowledge, suggests that cultural and experiential factors outweigh formal education globally, supporting our call for evidence-based educational interventions that address both knowledge deficits and attitude formation.

In summary, our obtained results emphasize the necessity of curriculum development and targeted educational interventions, particularly in non-health faculties, that should involve personal and direct experience to improve knowledge and safe use of DS and CAM. Recent studies indeed demonstrated that targeted DS/CAM education significantly improved knowledge and attitudes in nursing students and oncology healthcare providers [69,70].

### 4.5. Limitations of the Study

We need to address some limitations of the presented study, like self-reported data (e.g., self-assessment of knowledge), uneven distribution of study majors, and limited generalizability to other populations and regions. In particular, relying solely on self-reported data introduces potential biases, such as, for example, recall bias, misreporting of DS/CAM usage, or social desirability. This may lead to over- or underestimation of attitudes. Future studies may strengthen validity by combining self-reporting with objective measures, i.e., verified purchase records, clinical assessments, or observational data. In addition, our sample was convenience-based, as we only had two universities and not all universities in Croatia and Slovenia. Due to socio-cultural differences between Slovenia and Croatia, it was accordingly not possible to combine the student samples from health studies at universities in Slovenia and Croatia. Croatia and Slovenia are both post-transition European countries with a shared history. Their health education systems and public health environments have a common historical background but may differ in ways that are relevant to DS and CAM use. In particular, Slovenia entered the EU earlier than Croatia and did not have a long-lasting war on its territory as Croatia did. This allowed for a more systematic integration of public health promotion and evidence-based health education into university curricula of the health-focused programs. On the other side, Croatia’s implementation was objectively more variable. Distinct learning environments and cultural expectations around DS and CAM may accordingly arise. We did not evaluate the contextual contrast between the countries directly and may not elaborate upon it in the interpretation of the study’s comparative results. In addition, knowledge tests can be used to identify factual recall but may not necessarily reflect clinical decision-making or counseling ability. Finally, this was not an interventional study, but it can be used to propose some curriculum changes that need to be measured systematically.

## 5. Conclusions

The presented cross-national study, based on a combined assessment of a DS and CAM study, shows that dietary supplements (DS) and complementary and alternative medicine (CAM) are widely used and generally viewed positively among health and non-health studies students in Croatia and Slovenia. Expectedly, and in line with similar published studies, factual knowledge remains moderate among participants in the study and varies significantly across the involved faculties. Health-focused studies students from Slovenia achieved the highest knowledge scores, but gaps were evident in questions addressing safety, dosage, and interactions. Non-health studies students from Croatia expectedly demonstrated the lowest scores, highlighting the importance of extending educational initiatives beyond health-related programs.

Attitudes toward CAM were mildly positive across all groups, yet these attitudes were more strongly associated with supplement usage than with the assessed knowledge levels. This suggests that personal experience and cultural factors play a greater role in shaping perceptions than formal education, which is aligned with personalized medicine trends. The reliance on informal information sources such as family, peers, and social media further emphasizes the need for structured, evidence-based education. We also point to public health risks of uninformed DS and CAM use beyond the gaps in knowledge and positive attitudes measured in our study. To promote safer and more informed practices, integration of DS and CAM content into university curricula is essential, alongside targeted workshops and public health initiatives. For more informative results, longitudinal studies may be used in the future as well to track knowledge and attitudes across training years and after curricular changes.

We accordingly propose that traditional didactic teaching alone may be insufficient and has to be revised. This assumption is based on the study result that attitudes toward DS and CAM are strongly shaped by personal experience and cultural context. An educational approach that combines evidence-based approaches, experiential learning, intercultural health literacy training (i.e., exploration of culturally shaped perceptions of terms “natural,” “safe,” or “effective”), and peer-led interventions to reinforce evidence-based reasoning may be used to bridge the gap between personal beliefs and evidence-based understanding.

In summary, the presented study provides important insights for the interested scientific community by cross-nationally comparing the health-focused and non-health-focused program students with their knowledge and attitudes towards DS and CAM. The observed results, pointing to a discrepancy between knowledge and personal use, show that attitudes and experience often outweigh formal education. This point should be addressed by targeted and specifically designed educational interventions, substantiating health literacy and evidence-based education strategies.

## Figures and Tables

**Table 1:** Participant characteristics relevant to study outcomes, including sociodemographic data and type of study details.

Variable	Health-Focused Study—Faculty of Health Studies, Rijeka n = 480	Non-Health-Focused Study—Faculty of Teacher Education, Rijeka n = 242	Health-Focused Study—Faculty of Health Sciences, Maribor n = 87	Total n = 809
	n (%) or M (SD)			
Age (years)	24.7 (7.8)	22.9 (7.4)	22.8 (3.5)	23.9 (7.4)
*Sex*				
Female	383 (47.3)	231 (28.6)	76 (9.4)	690 (85.3)
Male	93 (11.5)	7 (0.9)	11 (1.4)	111 (13.7)
Did not want to declare	4 (0.5)	4 (0.5)	0 (0.0)	
*Study*				
Bachelor	351 (43.4)	219 (27.1)	75 (9.3)	645 (79.7)
Master	129 (15.9)	23 (2.8)	12 (1.5)	164 (20.3)
*Type of study*				
Part-time study	211 (26.1)	40 (4.9)	0 (0.0)	251 (31.0)
Full-time study	269 (33.3)	105 (13.0)	87 (10.7)	461 (57.0)
Missing data	0 (0.0)	97 (12.0)	0 (0.0)	97 (12.0)
*Major (field of study)*				
Nursing	220 (27.2)	/	/	220 (27.2)
Midwifery	59 (7.3)	/	/	59 (7.3)
Physiotherapy	157 (19.4)	/	/	157 (19.4)
Radiological technology	44 (5.4)	/	/	44 (5.4)
Early and Preschool Education	/	242 (29.9)	/	242 (29.9)
Faculty of Health Sciences (Nursing), Maribor	/	/	87 (10.8)	87 (10.8)

Legend: M—arithmetic mean, SD—standard deviation.

**Table 2:** CAM attitude test outcomes and correlations with supplement consumption and attitudes towards CAM.

Variable	Usage Supplements	Attitude Towards CAM
**Total sample**		
Test %	0.076	0.12 **
Usage supplements	-	0.28 **
Health-focused study (Faculty of Health Studies—Rijeka)		
Test %	0.10 *	0.09
Usage supplements	-	0.25 **
Non-health-focused study (Faculty of Teacher Education—Rijeka)		
Test %	0.05	0.13 *
Usage supplements	-	0.28 **
Health-focused study (Faculty of Health Sciences—Maribor)		
Test %	0.01	0.15
Usage supplements	-	0.38 **

Legend: * *p* < 0.05, ** *p* < 0.001, Spearman rank correlation coefficient.

## Data Availability

The original contributions presented in this study are included in the article/Supplementary Material. Further inquiries can be directed to the corresponding author.

## Supplementary Materials

The following supporting information can be downloaded at: https://www.mdpi.com/article/10.3390/foods15010061/s1.

## Abbreviations

The following abbreviations are used in this manuscript:

DS	dietary supplements
CAM	complementary and alternative medicine
