# Supplementary material for: Knowledge of Dietary Supplements and Attitudes Towards Complementary Medicine Among University Students: A Cross-Sectional Study
Source: Foods. 2025 Dec 24;15(1):61. doi: 10.3390/foods15010061 (PMC12785271; doi:10.3390/foods15010061)
Supplement: Supplementary file 1 [file foods-15-00061-s001.zip › foods-3972200-supplementary.pdf]

Supplementary files 1-3.

Supplementary material S1. Questionaries in Croatian

1. UPITNIK: ZNANJE O DODACIMA PREHRANI (CROATIAN)

Poštovani/poštovana,

Prema Direktivi Europske unije (46/2002), dodaci prehrani se definiraju kao proizvodi od koncentriranih izvora vitamina, minerala ili drugih tvari koje imaju prehrambeni ili fiziološki učinak, a namijenjeni su kao nadopuna uobičajenoj prehrani u cilju održavanja zdravlja. Najčešće se u tu svrhu koriste vitamini, minerali, aminokiseline, vlakna, esencijalne masne kiseline, biljni ekstrakti, alge, jestive gljive i ekstrakti biljaka. S obzirom na vrstu pripravka u kojem su dodaci prehrani dostupni na tržištu, razlikujemo kapsule, tablete, tekuće koncentrate u bočicama sa kapaljkom, vrećice sa praškom i slične formulacije. Molimo Vas stoga da pažljivo pročitate postavljena pitanja na temu poznavanja utjecaja dodataka prehrani na zdravlje i ljudi i odgovorite na postavljeno pitanje zaokruživanjem odgovora 'Točno' i 'Netočno'.

POTREBNO JE ZAOKRUŽITI JEDAN ODGOVOR VEZAN UZ PONUĐENU TVRDNJU:
TOČNO ILI NETOČNO

TOČNO ILI NETOČNO

---

1. Prije stavljanja na tržište, učinkovitost i sigurnost dodataka prehrani mora se testirati.

   TOČNO / NETOČNO

2. Sastojak može biti u prodaji kao lijek i dodatak prehrani.

   TOČNO / NETOČNO

3. Kvaliteta dodataka prehrani se rutinski testira prije stavljanja na tržište.

   TOČNO / NETOČNO

4. Pakiranje u kojem se nalaze dodaci prehrani mora imati istaknute informacije o mogućim neželjenim učincima uporabe dodataka prehrani.

   TOČNO / NETOČNO

5. Dodaci prehrani su hrana.

   TOČNO / NETOČNO

6. Registracija dodataka prehrani zahtijeva određivanje sastava proizvoda od strane nadležnog tijela.

   TOČNO / NETOČNO

7. Sigurnost svih dodataka prehrani u ljekarnama je testirana.

   TOČNO / NETOČNO

8. Uzimanje vitaminskih i mineralnih dodataka prehrani sprječava bolesti u zdravih ljudi.

TOČNO / NETOČNO

9. Uzimanje vitamina D u osoba starije životne dobi smanjuje rizik prijeloma kostiju.

TOČNO / NETOČNO

10. Uporaba preparata s magnezijem sprječava mišićne grčeve u osoba starije životne dobi.

TOČNO / NETOČNO

11. Uzimanje dodataka prehrani s kalcijem smanjuje rizik od prijeloma kostiju u osoba starije životne dobi.

TOČNO / NETOČNO

12. Uporaba multivitaminskih pripravaka štiti od bolesti srca.

TOČNO / NETOČNO

13. Uporaba antioksidansa sprječava razvoj raka.

TOČNO / NETOČNO

14. Redovita uporaba vitamina C umanjuje rizik od prehlade.

TOČNO / NETOČNO

15. Uzimanje prekomjerne količine suplemenata s magnezijem može uzrokovati proljev i mučninu.

TOČNO / NETOČNO

16. Vitamin C, prirodno prisutan u hrani, bolje se apsorbira od sintetičkog.

TOČNO / NETOČNO

17. Osobe s bolešću bubrega ne bi smjele uzimati vitamin C u visokim dozama.

TOČNO / NETOČNO

2. UPITNIK: STAVOVI PREMA KOMPLEMENTARNOJ I ALTERNATIVNOJ MEDICINI (KAM) (CROATIAN)

SPREMNOST ZA KORIŠTENJE KOMPLEMENTARNE I ALTERNATIVNE MEDICINE (KAM)

---

1. **Molimo Vas, označite svoj spol:**
   (označite samo jedno polje)

◯ muški

◯ ženski

◯ ne želim se izjasniti

2. **Upišite svoju dob u godinama:** __________

3. **Upišite svoj smjer studija na FZSRI/UFRI**
   (označite samo jedno polje)

____

4. **Na kojoj ste godini studija?**
(označite samo jedno polje)

◯ 1. godina preddiplomskog studija
◯ 2. godina preddiplomskog studija
◯ 3. godina preddiplomskog studija
◯ 1. godina diplomskog studija
◯ 2. godina diplomskog studija

PRED VAMA SE NALAZE DEFINICIJE KOMPLEMENTARNE I ALTERNATIVNE MEDICINE (KAM). MOLIMO VAS DA IH PROČITATE TE ODGOVORITE NA POSTAVLJENA PITANJA

- Komplementarna i alternativna medicina (KAM) je skupina različitih medicinskih i zdravstvenih sustava, praksi i proizvoda koji nisu dijelom konvencionalne medicine, ali se koriste zajedno s njom ili umjesto nje.

- Komplementarna medicina odnosi se na postupke liječenja koji se koriste zajedno s konvencionalnom medicinom i nadopunjuju ih.

- Alternativna medicina obuhvaća načine liječenja koji se primjenjuju kao zamjena za postupke konvencionalne medicine.

Korištenje metoda komplementarne i alternativne medicine *

Molimo Vas, za svaku od metoda KAM-a označite koristite li je ili jeste li je koristili u posljednjih godinu dana? (*u svakom redu označite samo jedno polje*)

	NIKAD	RIJETKO (1-2 puta godišnje)	POVREMENO (3-4 puta godišnje)	ČESTO (1-2 puta mjesečno)	VRLO ČESTO (1 ili više puta tjedno)
**1. ljekovito bilje**	◯	◯	◯	◯	◯
**2. vitamini i minerali**	◯	◯	◯	◯	◯
**3. probiotici**	◯	◯	◯	◯	◯
**4. ostali dodaci prehrani**	◯	◯	◯	◯	◯
**5. posebne dijete**	◯	◯	◯	◯	◯
**6. biofeedback**	◯	◯	◯	◯	◯

	NIKAD	RIJETKO (1-2 puta godišnje)	POVREMENO (3-4 puta godišnje)	ČESTO (1-2 puta mjesečno)	VRLO ČESTO (1 ili više puta tjedno)
**7. vođena imaginacija**	◯	◯	◯	◯	◯
**8. hipnoza**	◯	◯	◯	◯	◯
**9. meditacija**	◯	◯	◯	◯	◯
**10. vježbe dubokog disanja**	◯	◯	◯	◯	◯
**11. duhovnost i molitva**	◯	◯	◯	◯	◯
**12. ekspresivne art terapije**	◯	◯	◯	◯	◯
**13. progresivna mišićna relaksacija**	◯	◯	◯	◯	◯
**14. joga**	◯	◯	◯	◯	◯
**15. tai chi**	◯	◯	◯	◯	◯
**16. kiropraktika**	◯	◯	◯	◯	◯
**17. osteopatija**	◯	◯	◯	◯	◯
**18. masaža**	◯	◯	◯	◯	◯
**19. akupunktura**	◯	◯	◯	◯	◯
**20. refleksologija**	◯	◯	◯	◯	◯
**21. reiki**	◯	◯	◯	◯	◯
**22. Terapije pokretom (Feldenkreis metoda/ Pilates/ Alexander tehnika/ Rolfing metoda/ Trager tehnika/ drugo)**	◯	◯	◯	◯	◯
**23. qi gong**	◯	◯	◯	◯	◯
**24. iscjeljivanje dodirom**	◯	◯	◯	◯	◯
**25. elektromagnetska terapija**	◯	◯	◯	◯	◯
**26. tradicionalni iscjeljitelji**	◯	◯	◯	◯	◯
**27. Ayurveda**	◯	◯	◯	◯	◯

	NIKAD	RIJETKO (1-2 puta godišnje)	POVREMENO (3-4 puta godišnje)	ČESTO (1-2 puta mjesečno)	VRLO ČESTO (1 ili više puta tjedno)
**28. tradicionalna kineska medicina**	○	○	○	○	○
**29. naturopatija**	○	○	○	○	○
**30. homeopatija**	○	○	○	○	○
**31. aromaterapija**	○	○	○	○	○

PAŽLJIVO PROČITAJTE SVAKU TVRDNJU I POTOM OZNAČITE KOLIKO SE ODREĐENA TVRDNJA ODNOSI NA VAS

1 - u potpunosti se ne slažem;
2 - ne slažem se
3 - niti se slažem, niti se ne slažem
4 - slažem se
5 - u potpunosti se slažem

Zaokružite samo jedan broj pored svake tvrdnje.

**1. KAM tretmani pozitivno utječu na liječenje bolesti.**	**1**	**2**	**3**	**4**	**5**
**2. Liječenje bolesnika ne bi trebalo uključivati KAM.**	**1**	**2**	**3**	**4**	**5**
**3. KAMsadrži ideje i metode od kojih konvencionalna medicina može imati koristi.**	**1**	**2**	**3**	**4**	**5**
**4. Znanje o KAM važno mi je kao (budućem) zdravstvenom djelatniku.**	**1**	**2**	**3**	**4**	**5**
**5. KAM ne bih preporučila/o svojim bližnjima.**	**1**	**2**	**3**	**4**	**5**
**6. Zainteresiran/a sam za edukaciju o KAM metodama.**	**1**	**2**	**3**	**4**	**5**
**7. Uloga KAM-a u očuvanju zdravlja nije značajna.**	**1**	**2**	**3**	**4**	**5**
**8. KAM bih preporučila/o svojim pacijentima.**	**1**	**2**	**3**	**4**	**5**
**9. Farmaceutski lijekovi su učinkovitiji u liječenju od prirodnih lijekova.**	**1**	**2**	**3**	**4**	**5**
**10. Zdravstveni djelatnici bi trebali imati osnovno znanje o KAM.**	**1**	**2**	**3**	**4**	**5**

Supplementary material S2. Questionaries in Slovenian

1. VPRAŠALNIK: ZNANJE O PREHRANSKIH DOPOLNILIH (SLOVENIAN)

Spoštovani/Spoštovana,

V skladu z Direktivo Evropske unije (46/2002) se prehranska dopolnila opredeljujejo kot izdelki iz koncentriranih virov vitaminov, mineralov ali drugih snovi s prehranskim ali fiziološkim učinkom, namenjeni dopolnjevanju običajne prehrane z namenom ohranjanja zdravja. Najpogosteje se v ta namen uporabljajo vitamini, minerali, aminokisline, vlaknine, esencialne maščobne kisline, rastlinski izvlečki, alge, užitne gobe in izvlečki rastlin. Glede na vrsto pripravka, v katerem so prehranska dopolnila na voljo na trgu, razlikujemo kapsule, tablete, tekoče koncentrate v stekleničkah s kapalno pipeto, vrečke s praškom in podobne formulacije. Zato vas vljudno prosimo, da pozorno preberete zastavljena vprašanja na temo poznavanja vpliva prehranskih dopolnil na zdravje ljudi ter na postavljena vprašanja odgovorite tako, da obkrožite odgovor 'Točno' ali 'Netočno'.

Prosim označite podano trditev kot: PRAVILNO ali NEPRAVILNO

---

18. Preden prehranska dopolnila pridejo na trg, je treba preizkusiti njihovo učinkovitost in varnost.

PRAVILNO / NEPRAVILNO

19. Posamezna sestavina je lahko v prodaji kot zdravilo ali kot prehransko dopolnilo.

PRAVILNO / NEPRAVILNO

20. Kakovost prehranskih dopolnil se rutinsko testira pred prihodom na trg.

PRAVILNO / NEPRAVILNO

21. Embalaža, v kateri se nahajajo prehranska dopolnila, mora vsebovati jasno navedene informacije o možnih neželenih učinkih uporabe prehranskih dopolnil.

PRAVILNO / NEPRAVILNO

22. Prehranska dopolnila so živilo.

PRAVILNO / NEPRAVILNO

23. Registracija prehranskih dopolnil zahteva določitev sestave izdelka s strani pristojnega organa.

PRAVILNO / NEPRAVILNO

24. Varnost vseh prehranskih dopolnil v lekarnah je testirana.

PRAVILNO / NEPRAVILNO

25. Uživanje vitaminskih in mineralnih prehranskih dopolnil preprečuje bolezni pri zdravih ljudeh.

PRAVILNO / NEPRAVILNO

26. Uživanje vitamina D pri starejših osebah zmanjšuje tveganje za zlome kosti.

PRAVILNO / NEPRAVILNO

27. Uporaba pripravkov z magnezijem preprečuje mišične krče pri starejših osebah.

PRAVILNO / NEPRAVILNO

28. Uživanje prehranskih dopolnil s kalcijem zmanjšuje tveganje za zlome kosti pri starejših osebah.

PRAVILNO / NEPRAVILNO

29. Uporaba multivitaminskih pripravkov ščiti pred boleznimi srca.

PRAVILNO / NEPRAVILNO

30. Uporaba antioksidantov preprečuje razvoj raka.

PRAVILNO / NEPRAVILNO

31. Redna uporaba vitamina C zmanjšuje tveganje za prehlad.

PRAVILNO / NEPRAVILNO

32. Uživanje prekomerne količine prehranskih dopolnil z magnezijem lahko povzroči drisko in slabost.

PRAVILNO / NEPRAVILNO

33. Vitamin C, ki je naravno prisoten v hrani, se bolje absorbira kot sintetični.

PRAVILNO / NEPRAVILNO

34. Osebe z boleznijo ledvic ne bi smele uživati vitamina C v visokih odmerkih.

PRAVILNO / NEPRAVILNO

2. VPRAŠALNIK: ODNOS DO KOMPLEMENTARNE IN ALTERNATIVNE MEDICINE (CAM) (SLOVENIAN)

PRIPRAVLJENOST ZA UPORABO KOMPLEMENTARNE IN ALTERNATIVNE MEDICINE (KAM)

1. **Prosim označite vaš spol:**
   (označite samo eno polje)
   - o moški
   - o ženska
   - o ne želim se opredeliti

5. **Vpišite svojo starost v letih:** __________

6. **Označite svojo študijsko smer na UM FZV:**
   (označite samo eno polje)
   - o Dodiplomski študij Zdravstvene nege.
   - o Podiplomski študij Zdravstvene nege, smer Zdravstvena nega.
   - o Podiplomski študij Zdravstvene nege, smer Preventivna in klinična prehrana.
   - o Podiplomski študij Zdravstvene nege, smer Urgentna stanja v zdravstvu.
   - o Podiplomski študij Zdravstvene nege, smer Integrirana obravnava kroničnih bolnikov v napredni zdravstveni negi.

- Podiplomski študij Zdravstvene nege, smer Mentalno zdravje v napredni praksi zdravstvene nege.
- Podiplomski študij Management v zdravstvu in socialnem varstvu.

7. **V katerem letniku študija ste?**

(označite samo eno polje)

- leto dodiplomskega študija
- leto dodiplomskega študija
- leto dodiplomskega študija
- leto podiplomskega študija
- leto podiplomskega študija

PRED VAMI SO DEFINICIJE KOMPLEMENTARNE IN ALTERNATIVNE MEDICINE (KAM). PROSIMO, DA JIH PREBERETE IN ODGOVORITE NA POSTAVLJENA VPRAŠANJA

Komplementarna in alternativna medicina (KAM) je skupina različnih medicinskih in zdravstvenih sistemov, praks in izdelkov, ki niso del konvencionalne medicine, vendar se uporabljajo skupaj z njo ali namesto nje.

- ❖ Komplementarna medicina se nanaša na metode zdravljenja, ki se uporabljajo skupaj s konvencionalno medicino in jo dopolnjujejo.
- ❖ Alternativna medicina zajema načine zdravljenja, ki se uporabljajo kot nadomestilo za metode konvencionalne medicine.

UPORABA METOD KOMPLEMENTARNE IN ALTERNATIVNE MEDICINE

Prosimo, da za vsako metodo KAM označite, ali jo uporabljate ali ste jo uporabljali v zadnjem letu. *(V vsaki vrstici označite samo eno polje)*

	NIKOLI	REDKO (1-2 krat letno)	OBČASNO (3-4 krat letno)	POGOSTO (1-2 krat mesečno)	ZELO POGOSTO (1 ali večkrat tedensko)
32. Zdravilna zelišča	○	○	○	○	○
33. Vitamini in minerali	○	○	○	○	○
34. Probiotiki	○	○	○	○	○
35. Drugi prehranski dodatki	○	○	○	○	○
36. Posebne diete	○	○	○	○	○
37. Biofeedback	○	○	○	○	○
38. Vodena imaginacija	○	○	○	○	○

	NIKOLI	REDKO (1-2 krat letno)	OBČASNO (3-4 krat letno)	POGOSTO (1-2 krat mesečno)	ZELO POGOSTO (1 ali večkrat tedensko)
39. Hipnoza	○	○	○	○	○
40. Meditacija	○	○	○	○	○
41. Vaje za globoko dihanje	○	○	○	○	○
42. Duhovnost in molitev	○	○	○	○	○
43. Ekspresivne umetniške terapije	○	○	○	○	○
44. Progresivna mišična relaksacija	○	○	○	○	○
45. Joga	○	○	○	○	○
46. Tai chi	○	○	○	○	○
47. Kiropraktika	○	○	○	○	○
48. Osteopatija	○	○	○	○	○
49. Masaža	○	○	○	○	○
50. Akupunktura	○	○	○	○	○
51. Refleksoterapija	○	○	○	○	○
52. Reiki	○	○	○	○	○
53. Gibalne terapije (Feldenkraisova metoda, pilates, Alexander tehnika, Rolfing metoda, Trager tehnika, drugo)	○	○	○	○	○
54. Qi gong	○	○	○	○	○
55. Zdravljenje z dotikom	○	○	○	○	○
56. Elektromagnetna terapija	○	○	○	○	○
57. Tradicionalno zdravilstvo	○	○	○	○	○
58. Ajurveda	○	○	○	○	○
59. Tradicionalna kitajska medicina	○	○	○	○	○
60. Naturopatija	○	○	○	○	○

	NIKOLI	REDKO (1-2 krat letno)	OBČASNO (3-4 krat letno)	POGOSTO (1-2 krat mesečno)	ZELO POGOSTO (1 ali večkrat tedensko)
61. Homeopatija	○	○	○	○	○
62. Aromaterapija	○	○	○	○	○

NATANČNO PREBERETE VSAKO TRDITEV IN NATO OZNAČITE, KAKO SE DOLOČENA TRDITEV NANAŠA NA VAS.

1 - popolnoma se ne strinjam
2 - se ne strinjam
3 - niti se strinjam, niti se ne strinjam
4 - se strinjam
5 - popolnoma se strinjam

Obkrožite samo eno od števil ob vsaki trditvi.

1. KAM tretmaji pozitivno vplivajo na zdravljenje bolezni.	**1**	**2**	**3**	**4**	**5**
2. Zdravljenje bolnikov ne bi smelo vključevati KAM.	**1**	**2**	**3**	**4**	**5**
3. KAM vsebuje ideje in metode, od katerih lahko konvencionalna medicina ima korist.	**1**	**2**	**3**	**4**	**5**
4. Znanje o KAM je pomembno zame kot bodočega zdravstvenega delavca.	**1**	**2**	**3**	**4**	**5**
5. KAM ne bi priporočil/a svojim bližnjim.	**1**	**2**	**3**	**4**	**5**
6. Zanimam se za izobraževanje o metodah KAM.	**1**	**2**	**3**	**4**	**5**
7. Vloga KAM pri ohranjanju zdravja ni pomembna.	**1**	**2**	**3**	**4**	**5**
8. KAM bi priporočil/a svojim pacientom.	**1**	**2**	**3**	**4**	**5**
9. Farmacevtska zdravila so učinkovitejša pri zdravljenju kot naravna zdravila.	**1**	**2**	**3**	**4**	**5**
10. Zdravstveni delavci bi morali imeti osnovno znanje o KAM.	**1**	**2**	**3**	**4**	**5**

Zahvaljujemo se za vaš čas.

Supplementary material S3. Questionaries in English.

1. QUESTIONNAIRE: KNOWLEDGE ABOUT DIETARY SUPPLEMENTS

Dear Sir/Madam,

According to the European Union Directive (46/2002), dietary supplements are defined as products derived from concentrated sources of vitamins, minerals, or other substances with a nutritional or physiological effect, intended to supplement the normal diet to maintain health. Vitamins, minerals, amino acids, fibers, essential fatty acids, herbal extracts, algae, edible mushrooms, and plant extracts are most commonly used for this purpose. Depending on the type of formulation in which dietary supplements are available on the market, we distinguish capsules, tablets, liquid concentrates in dropper bottles, sachets with powder, and similar forms. Therefore, we kindly ask you to carefully read the questions regarding knowledge of the effects of dietary supplements on human health and answer each question by circling either 'True' or 'False'.

PLEASE CIRCLE ONE ANSWER FOR EACH STATEMENT:

TRUE OR FALSE:

---

1. Before being placed on the market, the efficacy and safety of dietary supplements must be tested.

TRUE / FALSE

2. An ingredient can be sold both as a medicine and as a dietary supplement.

TRUE / FALSE

3. The quality of dietary supplements is routinely tested before being placed on the market.

TRUE / FALSE

4. Packaging of dietary supplements must clearly display information about possible adverse effects of use.

TRUE / FALSE

5. Dietary supplements are considered food.

TRUE / FALSE

6. Registration of dietary supplements requires determination of the product's composition by the competent authority.

TRUE / FALSE

7. The safety of all dietary supplements available in pharmacies has been tested.

TRUE / FALSE

8. Taking vitamin and mineral supplements prevents diseases in healthy individuals.

TRUE / FALSE

9. Taking vitamin D in older adults reduces the risk of bone fractures.

TRUE / FALSE

10. Use of magnesium supplements prevents muscle cramps in older adults.

TRUE / FALSE

11. Taking calcium supplements reduces the risk of bone fractures in older adults.

TRUE / FALSE

12. Use of multivitamin preparations protects against heart disease.

TRUE / FALSE

13. Use of antioxidants prevents the development of cancer.

TRUE / FALSE

14. Regular use of vitamin C reduces the risk of colds.

TRUE / FALSE

15. Taking excessive amounts of magnesium supplements can cause diarrhea and nausea.

TRUE / FALSE

16. Vitamin C naturally present in food is better absorbed than synthetic vitamin C.

TRUE / FALSE

17. People with kidney disease should not take high doses of vitamin C.

TRUE / FALSE

2. QUESTIONNAIRE: ATTITUDE TOWARDS COMPLEMENTARY AND ALTERNATIVE MEDICINE (CAM)

READINESS TO USE COMPLEMENTARY AND ALTERNATIVE MEDICINE (CAM)

---

1. Please indicate your gender:

(Please select only one option)

◯ Male

◯ Female

◯ Prefer not to say

Enter your age in years: __________

Enter your field of study at FZSRI/UFRI:

(Please select only one option)

____

2. Which year of study are you currently in?

(Please select only one option)

◯ 1st year of undergraduate study

◯ 2nd year of undergraduate study

◯ 3rd year of undergraduate study

◯ 1st year of graduate study

◯ 2nd year of graduate study

BELOW ARE DEFINITIONS OF COMPLEMENTARY AND ALTERNATIVE MEDICINE (CAM). PLEASE READ THEM CAREFULLY AND ANSWER THE FOLLOWING QUESTIONS

- Complementary and Alternative Medicine (CAM) is a group of various medical and health systems, practices, and products that are not part of conventional medicine, but are used alongside or instead of it.
- Complementary medicine refers to treatment practices used together with conventional medicine to complement it.
- Alternative medicine includes treatment methods applied as a substitute for conventional medical procedures.

Use of Complementary and Alternative Medicine Methods *

For each CAM method listed below, please indicate whether you are currently using it or have used it in the past year. (Please select only one option in each row)

	NEVER	RARELY (1–2 times per year)	OCCASIONALLY (3–4 times per year)	OFTEN (1–2 times per month)	VERY OFTEN (1 or more times per week)
**63. Medicinal herbs**	○	○	○	○	○
**64. Vitamins and minerals**	○	○	○	○	○
**65. Probiotics**	○	○	○	○	○
**66. Other dietary supplements**	○	○	○	○	○
**67. Special diets**	○	○	○	○	○
**68. Biofeedback**	○	○	○	○	○
**69. Guided imagery**	○	○	○	○	○
**70. Hypnosis**	○	○	○	○	○
**71. Meditation**	○	○	○	○	○
**72. Deep breathing exercises**	○	○	○	○	○
**73. Spirituality and prayer**	○	○	○	○	○
**74. Expressive art therapies**	○	○	○	○	○
**75. Yoga**	○	○	○	○	○

	NEVER	RARELY (1–2 times per year)	OCCASIONALLY (3–4 times per year)	OFTEN (1–2 times per month)	VERY OFTEN (1 or more times per week)
**76. Tai Chi**	◯	◯	◯	◯	◯
**77. Spirituality and prayer**	◯	◯	◯	◯	◯
**78. Chiropractic**	◯	◯	◯	◯	◯
**79. Osteopathy**	◯	◯	◯	◯	◯
**80. Massage**	◯	◯	◯	◯	◯
**81. Acupuncture**	◯	◯	◯	◯	◯
**82. Reflexology**	◯	◯	◯	◯	◯
**83. Reiki**	◯	◯	◯	◯	◯
**84. Movement therapies (Feldenkrais method / Pilates / Alexander technique / Rolfing method / Trager approach / other)**	◯	◯	◯	◯	◯
**85. Qi Gong**	◯	◯	◯	◯	◯
**86. Healing touch**	◯	◯	◯	◯	◯
**87. Electromagnetic therapy**	◯	◯	◯	◯	◯
**88. Traditional healers**	◯	◯	◯	◯	◯
**89. Ayurveda**	◯	◯	◯	◯	◯
**90. Traditional Chinese medicine**	◯	◯	◯	◯	◯
**91. Naturopathy**	◯	◯	◯	◯	◯
**92. Homeopathy**	◯	◯	◯	◯	◯
**93. Aromatherapy**	◯	◯	◯	◯	◯

PLEASE READ EACH STATEMENT CAREFULLY AND THEN INDICATE TO WHAT EXTENT THE STATEMENT APPLIES TO YOU.

1 – Strongly disagree
2 – Disagree
3 – Neither agree nor disagree
4 – Agree
5 – Strongly agree

(Please circle only one number next to each statement).

**11. CAM treatments have a positive effect on disease treatment.**	**1**	**2**	**3**	**4**	**5**
**12. Patient care should not include CAM.**	**1**	**2**	**3**	**4**	**5**
**13. CAM contains ideas and methods from which conventional medicine can benefit.**	**1**	**2**	**3**	**4**	**5**
**14. Knowledge of CAM is important to me as a (future) healthcare professional.**	**1**	**2**	**3**	**4**	**5**
**15. I would not recommend CAM to my family or close ones.**	**1**	**2**	**3**	**4**	**5**
**16. I am interested in education on CAM methods.**	**1**	**2**	**3**	**4**	**5**
**17. The role of CAM in maintaining health is not significant.**	**1**	**2**	**3**	**4**	**5**
**18. I would recommend CAM to my patients.**	**1**	**2**	**3**	**4**	**5**
**19. Pharmaceutical medicines are more effective in treatment than natural remedies.**	**1**	**2**	**3**	**4**	**5**
**20. Healthcare professionals should have basic knowledge of CAM.**	**1**	**2**	**3**	**4**	**5**

Supplementary material S4. Test results according to the faculty.

Variable	Health Focused study - Faculty of health studies – Rijeka n=480	Non-health Focused study - Faculty of Teacher Education - Rijeka n=242	Health Focused study - Faculty of Health Sciences – Maribor n=87	total n=809	P[1]
	n (%) or M (SD)				
Percentage of correct answers	72.4 (13.2)**	68.8 (14.1)	71.8 (17.1)	71.2 (14.0)	0.005*
Test pass					
Failed (<9 answers)	26 (5.4)	21 (8.7)	8 (9.2)	55 (6.8)	0.167
Passed (>8 answers)	454 (94.6)	221 (91.3)	79 (90.8)	754 (93.2)	
Grades					
1 (<50%)	26 (5.4)	21 (8.7)	8 (9.2)	55 (6.8)	
2 (50-64.99%)	64 (13.3)	43 (17.8)	16 (18.4)	123 (15.2)	0.010
3 (65-74.99%)	157 (32.7)	86 (35.5)	23 (26.4)	266 (32.9)	
4 (75-89.99%)	203 (42.3)	80 (33.1)	28 (32.2)	311 (38.4)	
5 (>90%)	30 (6.3)	12 (5.0)	12 (13.8)	54 (6.7)	
Correct answers (questions from (37)*)*					
Q1 -Before being marketed, dietary supplements must be tested for efficacy and safety.	471 (98.1)	240 (99.2)	83 (95.4)	794 (98.1)	0.082
Q2 - An ingredient may be sold both as a medicine or as a dietary supplement.	400 (83.3)	193 (79.8)	69 (79.3)	662 (81.8)	0.406

Q3 - The quality of dietary supplements is routinely tested before being marketed.	421 (87.9)	213 (88.4)	66 (75.9)	700 (86.7)	0.007
Q4 - The packaging of dietary supplements must contain information on possible adverse effects resulting from their use.	443 (92.3)	231 (95.9)	74 (85.1)	748 (92.6)	0.004
Q5 - Dietary supplements are food.					
Q6 - Dietary supplement registration requires assessing the composition of the product by the appropriate supervisory body.	50 (10.4)	20 (8.3)	26 (29.9)	96 (11.9)	< 0.001
Q7 - All dietary supplements sold in pharmacies have been tested for safety.	445 (93.3)	223 (92.1)	71 (81.6)	739 (91.7)	0.001
Q8 - Taking vitamin and mineral supplements prevents diseases in healthy people.	368 (77.1)	193 (80.1)	65 (75.6)	626 (77.9)	0.580
Q9 - In the elderly, taking vitamin D reduces the risk of bone fractures.	175 (36.5)	93 (38.6)	40 (46.0)	308 (38.2)	0.246
Q10 - In the elderly, the use of magnesium preparations prevents muscle cramps.	381 (79.5)	148 (61.4)	71 (81.6)	600 (74.3)	< 0.001
Q11 - Taking dietary supplements containing calcium reduces the risk of bone fractures in the elderly.	427 (89.0)	199 (82.6)	75 (86.2)	701 (86.8)	0.057

Q12 - The use of multivitamin preparations protects against heart diseases.	402 (83.8)	177 (73.4)	75 (86.2)	654 (80.9)	0.002
Q13 - The use of antioxidants prevents the development of cancer.	140 (29.2)	51 (21.3)	28 (32.2)	219 (27.2)	0.046
Q14 - Regular use of vitamin C reduces the risk of catching a cold.	174 (36.3)	67 (27.9)	39 (44.8)	280 (34.7)	0.009
Q15 - Taking excessive amounts of magnesium supplements can cause diarrhea and nausea.	418 (87.1)	216 (89.6)	69 (79.3)	703 (87.0)	0.049
Q16 - Vitamin C naturally present in food is better assimilated than synthetic.	441 (91.9)	219 (90.9)	82 (94.3)	742 (91.8)	0.613
Q17 - People with kidney disease should not use high doses of vitamin C.	391 (81.5)	195 (80.9)	62 (71.3)	648 (80.2)	0.085
	358 (75.5)	161 (67.9)	67 (77.9)	586 (73.5)	0.060

Legend: 1- all tests were $\chi 2$ tests except for the Percentage of correct answers, *- one-way ANOVA, ** - post hoc Newman Keuls test: difference $p<0.05$ between Faculty of Health sciences – Maribor and Faculty of Teacher Education – Rijeka
